# 1-*sec*-Butyl-3-[hy­droxy(1-methyl-1*H*-indol-3-yl)methyl­idene]pyrrolidine-2,4-dione

**DOI:** 10.1107/S1600536810030679

**Published:** 2010-08-11

**Authors:** Hai-zhen Xu, You-Quan Zhu

**Affiliations:** a College of Chemistry, Tianjin Normal University, 393 Binshuixi Road, Xiqing District, Tianjin 300387, People’s Republic of China; b State Key Laboratory of Elemento-Organic Chemistry, Nankai University, Tianjin 300071, People’s Republic of China

## Abstract

In the title compound, C_18_H_20_N_2_O_3_, the dihedral angle between the indole ring system (r.m.s. deviation = 0.018 Å) and the hy­droxy­methyl­enepyrrolidine-2,4-dione plane (r.m.s. deviation = 0.036 Å) is 9.87 (7)°. The keto and enol groups are involved in an intra­molecular O—H⋯O hydrogen bond. An intra­molecular C—H⋯O inter­action also occurs. The *sec*-butyl group is disordered over two orientations corresponding to an approximate 180° rotation about the N—C bond, with occupancies of 0.670 (6) and 0.330 (6). In the crystal, mol­ecules are linked into chains along the *c* axis by C—H⋯O hydrogen bonds.

## Related literature

For the anti­biotic activity of 3-acyl­pyrrolidine-2,4-dione compounds, see: Baan *et al.* (1978 ▶); Holzapfel *et al.* (1970 ▶); Mackellar *et al.* (1971 ▶); Matsuo *et al.* (1980 ▶); Rinehart *et al.* (1963 ▶); Sticking (1959 ▶); Wu *et al.* (2002 ▶). For a related structure, see: Ellis & Spek (2001 ▶). For bond-length data, see: Allen *et al.* (1987 ▶).
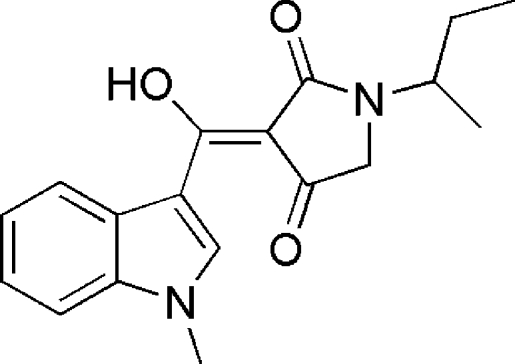


## Experimental

### 

#### Crystal data

C_18_H_20_N_2_O_3_
*M* *_r_* = 312.36
Monoclinic, 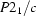

*a* = 11.781 (2) Å
*b* = 10.529 (2) Å
*c* = 12.644 (3) Å
β = 97.18 (3)°
*V* = 1556.1 (5) Å^3^
*Z* = 4
Mo *K*α radiation
μ = 0.09 mm^−1^
*T* = 113 K
0.18 × 0.16 × 0.10 mm

#### Data collection

Rigaku Saturn diffractometer
Absorption correction: multi-scan (*CrystalClear*; Rigaku, 2005 ▶) *T*_min_ = 0.984, *T*_max_ = 0.991
12618 measured reflections
3698 independent reflections
2687 reflections with *I* > 2σ(*I*)
*R*_int_ = 0.034

#### Refinement

*R*[*F* ^2^ > 2σ(*F* ^2^)] = 0.055
*wR*(*F* ^2^) = 0.168
*S* = 1.13
3698 reflections
218 parameters
10 restraints
H-atom parameters constrained
Δρ_max_ = 0.66 e Å^−3^
Δρ_min_ = −0.57 e Å^−3^

### 

Data collection: *CrystalClear* (Rigaku, 2005 ▶); cell refinement: *CrystalClear*; data reduction: *CrystalClear*; program(s) used to solve structure: *SHELXS97* (Sheldrick, 2008 ▶); program(s) used to refine structure: *SHELXL97* (Sheldrick, 2008 ▶); molecular graphics: *SHELXTL* (Sheldrick, 2008 ▶); software used to prepare material for publication: *SHELXTL*.

## Supplementary Material

Crystal structure: contains datablocks global, I. DOI: 10.1107/S1600536810030679/ci5139sup1.cif

Structure factors: contains datablocks I. DOI: 10.1107/S1600536810030679/ci5139Isup2.hkl

Additional supplementary materials:  crystallographic information; 3D view; checkCIF report

## Figures and Tables

**Table 1:** Hydrogen-bond geometry (Å, °)

*D*—H⋯*A*	*D*—H	H⋯*A*	*D*⋯*A*	*D*—H⋯*A*
O1—H1⋯O3	0.84	1.72	2.5003 (19)	154
C8—H8⋯O2	0.95	2.12	2.916 (2)	140
C9—H9*C*⋯O2^i^	0.98	2.51	3.441 (3)	159

Symmetry code: (i) 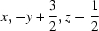.

**Fig. 1.**
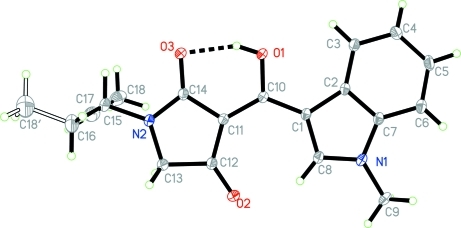
The molecular structure of the title compound, showing 30% probability displacement ellipsoids and the atom-numbering scheme. Both disorder components are shown.

## supplementary crystallographic information

### Comment

Many compounds containing the 3-acylpyrrolidine-2,4-dione moiety are novel heterocyclic compounds with antibiotic activity. Some of them are tenuazonic (Sticking, 1959), streptolydigin (Rinehart *et al.*, 1963), tirandamycin (Mackellar *et al.*, 1971), malonomycin (Baan *et al.*, 1978), alpha-cyclopiazonic acid (Sticking, 1959) and bata-cyclopiazonic acid (Holzapfel *et al.*, 1970). All these compounds possess a 3-acyltetramic acid moiety as a tricarbonylmethane structure and their hydrogen chemical shift of the enol hydroxy is about 11 p.p.m. (Wu *et al.*, 2002). On the other hand, most of the excellent inhibitors of *p*-hydroxyphenylpyruvate dioxygenase also possess similar characteristics, which are crucial for their bioactivity. Up to now, we have synthesized a series of 3-(un)substituted aroyl-1-benzylpyrrolidine-2,4- dione compounds and some of them have high herbicidal activity. The structure of the title compound, (I), helps us to investigate the relationship between structure and herbicidal activity.

The molecular structure of (I) is shown in Fig. 1. Atom H1, involved in intramolecular hydrogen bonding between O1 and O3, was assigned to O1 rather than to O3, based on bond lengths. The C14—O3 distance is 1.254 (2) Å, which is longer than the C12—O2 distance of 1.231 (2) Å. In contrast, the C10—O1 distance [1.322 (2) Å] is intermediate between the normal carbonyl bond and the C—O single bond length (Allen *et al.* 1987). A similar situation has been found in 3-(1-hydroxyethylidene)-1- phenylpyrrolidine-2,4-dione, which contains the same pyrrolidine skeleton (Ellis & Spek, 2001). The dihedral angle formed by the indole ring system (r.m.s. deviation 0.018 Å) and the hydroxymethylene-pyrrolidine-2,4-dione plane (r.m.s. deviation 0.036 Å) is 9.87 (7)°.

### Experimental

The title compound was obtained according to the reported procedure of Matsuo *et al.* (1980). Colourless single crystals were obtained by recrystallization of the title compound from petroleum ether and ethyl acetate.

### Refinement

The sec-butyl group is disordered over two orientations corresponding to an approximately 180° rotation about the N2—C15 bond, with refined occupancies of 0.670 (6) and 0.330 (6). All C—C distances in this group were restrained to 1.540 (5) Å. H atoms were placed in calculated positions, with C–H = 0.95–0.98 Å and O–H = 0.84 A°, and included in the final cycles of refinement using a riding model, with *U*_iso_(H) = 1.2U_eq_(C) and 1.5U_eq_(O).

### Crystal data

**Table:**

C_18_H_20_N_2_O_3_	*F*(000) = 664
*M**_r_* = 312.36	*D*_x_ = 1.333 Mg m^−^^3^
Monoclinic, *P*2_1_/*c*	Mo *K*α radiation, λ = 0.71073 Å
Hall symbol: -P 2ybc	Cell parameters from 4289 reflections
*a* = 11.781 (2) Å	θ = 2.5–27.9°
*b* = 10.529 (2) Å	µ = 0.09 mm^−^^1^
*c* = 12.644 (3) Å	*T* = 113 K
β = 97.18 (3)°	Prism, yellow
*V* = 1556.1 (5) Å^3^	0.18 × 0.16 × 0.10 mm
*Z* = 4	

### Data collection

**Table:**

Rigaku Saturn diffractometer	3698 independent reflections
Radiation source: fine-focus sealed tube	2687 reflections with *I* > 2σ(*I*)
graphite	*R*_int_ = 0.034
ω scans	θ_max_ = 27.9°, θ_min_ = 2.6°
Absorption correction: multi-scan (*CrystalClear*; Rigaku, 2005)	*h* = −9→15
*T*_min_ = 0.984, *T*_max_ = 0.991	*k* = −13→13
12618 measured reflections	*l* = −16→16

### Refinement

**Table:**

Refinement on *F*^2^	Primary atom site location: structure-invariant direct methods
Least-squares matrix: full	Secondary atom site location: difference Fourier map
*R*[*F*^2^ > 2σ(*F*^2^)] = 0.055	Hydrogen site location: inferred from neighbouring sites
*wR*(*F*^2^) = 0.168	H-atom parameters constrained
*S* = 1.13	*w* = 1/[σ^2^(*F*_o_^2^) + (0.0947*P*)^2^ + 0.1812*P*] where *P* = (*F*_o_^2^ + 2*F*_c_^2^)/3
3698 reflections	(Δ/σ)_max_ = 0.001
218 parameters	Δρ_max_ = 0.66 e Å^−^^3^
10 restraints	Δρ_min_ = −0.57 e Å^−^^3^

### Special details

**Table:**

Geometry. All e.s.d.'s (except the e.s.d. in the dihedral angle between two l.s. planes) are estimated using the full covariance matrix. The cell e.s.d.'s are taken into account individually in the estimation of e.s.d.'s in distances, angles and torsion angles; correlations between e.s.d.'s in cell parameters are only used when they are defined by crystal symmetry. An approximate (isotropic) treatment of cell e.s.d.'s is used for estimating e.s.d.'s involving l.s. planes.
Refinement. Refinement of *F*^2^ against ALL reflections. The weighted *R*-factor *wR* and goodness of fit *S* are based on *F*^2^, conventional *R*-factors *R* are based on *F*, with *F* set to zero for negative *F*^2^. The threshold expression of *F*^2^ > σ(*F*^2^) is used only for calculating *R*-factors(gt) *etc*. and is not relevant to the choice of reflections for refinement. *R*-factors based on *F*^2^ are statistically about twice as large as those based on *F*, and *R*- factors based on ALL data will be even larger.

### Fractional atomic coordinates and isotropic or equivalent isotropic displacement parameters (Å^2^)

**Table:**

	*x*	*y*	*z*	*U*_iso_*/*U*_eq_	Occ. (<1)
O1	0.66886 (10)	1.07272 (11)	0.42060 (10)	0.0232 (3)	
H1	0.6268	1.0643	0.4691	0.035*	
O2	0.93397 (11)	0.78344 (12)	0.54741 (11)	0.0297 (3)	
O3	0.59380 (11)	1.00771 (11)	0.58894 (10)	0.0258 (3)	
N1	0.98265 (12)	0.96858 (14)	0.26733 (12)	0.0224 (3)	
N2	0.69798 (14)	0.85187 (15)	0.68474 (13)	0.0282 (4)	
C1	0.83455 (14)	1.00283 (15)	0.35989 (13)	0.0194 (4)	
C2	0.81236 (14)	1.06960 (15)	0.25917 (13)	0.0202 (4)	
C3	0.72166 (16)	1.14252 (16)	0.20819 (15)	0.0253 (4)	
H3	0.6560	1.1595	0.2425	0.030*	
C4	0.72960 (18)	1.18928 (18)	0.10697 (15)	0.0306 (4)	
H4	0.6677	1.2368	0.0714	0.037*	
C5	0.82620 (19)	1.16822 (18)	0.05608 (16)	0.0319 (5)	
H5	0.8299	1.2041	−0.0123	0.038*	
C6	0.91688 (17)	1.09601 (17)	0.10329 (15)	0.0278 (4)	
H6	0.9827	1.0810	0.0687	0.033*	
C7	0.90694 (15)	1.04631 (16)	0.20424 (14)	0.0216 (4)	
C8	0.93954 (14)	0.94263 (16)	0.35943 (14)	0.0211 (4)	
H8	0.9757	0.8910	0.4154	0.025*	
C9	1.08481 (15)	0.91093 (18)	0.23351 (16)	0.0285 (4)	
H9A	1.1285	0.8681	0.2943	0.043*	
H9B	1.1322	0.9770	0.2065	0.043*	
H9C	1.0623	0.8489	0.1770	0.043*	
C10	0.75981 (14)	0.99897 (15)	0.44100 (13)	0.0186 (4)	
C11	0.76929 (14)	0.92599 (15)	0.53493 (14)	0.0201 (4)	
C12	0.84678 (15)	0.82736 (17)	0.57785 (14)	0.0236 (4)	
C13	0.80043 (16)	0.77699 (18)	0.67725 (16)	0.0298 (4)	
H13A	0.7816	0.6855	0.6696	0.036*	
H13B	0.8569	0.7890	0.7413	0.036*	
C14	0.67860 (15)	0.93502 (16)	0.60328 (14)	0.0220 (4)	
C15	0.61807 (18)	0.8307 (2)	0.76310 (17)	0.0412 (6)	
H15	0.5520	0.8898	0.7455	0.049*	
C16	0.6765 (2)	0.8655 (4)	0.87609 (19)	0.0717 (10)	
H16A	0.7046	0.9511	0.8762	0.108*	0.670 (6)
H16B	0.6218	0.8582	0.9260	0.108*	0.670 (6)
H16C	0.7391	0.8085	0.8962	0.108*	0.670 (6)
H16D	0.7043	0.9511	0.8756	0.086*	0.330 (6)
H16E	0.7411	0.8106	0.8945	0.086*	0.330 (6)
C17	0.5719 (2)	0.6974 (2)	0.7563 (2)	0.0582 (8)	
H17A	0.5187	0.6874	0.8073	0.070*	0.670 (6)
H17B	0.6336	0.6388	0.7749	0.070*	0.670 (6)
H17C	0.5373	0.6820	0.6846	0.087*	0.330 (6)
H17D	0.6328	0.6378	0.7745	0.087*	0.330 (6)
H17E	0.5155	0.6875	0.8043	0.087*	0.330 (6)
C18	0.5146 (3)	0.6638 (3)	0.6507 (3)	0.0524 (13)	0.670 (6)
H18A	0.4453	0.7153	0.6346	0.079*	0.670 (6)
H18B	0.4939	0.5736	0.6495	0.079*	0.670 (6)
H18C	0.5662	0.6800	0.5971	0.079*	0.670 (6)
C18'	0.5954 (8)	0.8519 (12)	0.9604 (7)	0.087 (4)	0.330 (6)
H18D	0.5255	0.9007	0.9387	0.130*	0.330 (6)
H18E	0.6326	0.8840	1.0287	0.130*	0.330 (6)
H18F	0.5758	0.7621	0.9678	0.130*	0.330 (6)

### Atomic displacement parameters (Å^2^)

**Table:**

	*U*^11^	*U*^22^	*U*^33^	*U*^12^	*U*^13^	*U*^23^
O1	0.0214 (6)	0.0279 (7)	0.0218 (7)	0.0049 (5)	0.0084 (5)	0.0028 (5)
O2	0.0252 (7)	0.0340 (7)	0.0313 (8)	0.0094 (6)	0.0096 (6)	0.0056 (6)
O3	0.0230 (6)	0.0314 (7)	0.0240 (7)	0.0073 (5)	0.0073 (5)	0.0040 (5)
N1	0.0202 (7)	0.0254 (7)	0.0229 (8)	−0.0016 (6)	0.0077 (6)	−0.0021 (6)
N2	0.0258 (8)	0.0336 (8)	0.0275 (8)	0.0063 (7)	0.0119 (7)	0.0098 (6)
C1	0.0201 (8)	0.0194 (8)	0.0193 (8)	−0.0042 (6)	0.0051 (7)	−0.0015 (6)
C2	0.0228 (8)	0.0190 (8)	0.0198 (8)	−0.0036 (6)	0.0063 (7)	−0.0012 (6)
C3	0.0251 (9)	0.0254 (9)	0.0268 (10)	−0.0003 (7)	0.0085 (8)	0.0016 (7)
C4	0.0356 (10)	0.0301 (9)	0.0265 (10)	0.0024 (8)	0.0062 (8)	0.0053 (8)
C5	0.0441 (12)	0.0315 (10)	0.0222 (9)	0.0004 (9)	0.0123 (9)	0.0059 (8)
C6	0.0333 (10)	0.0275 (9)	0.0248 (9)	−0.0033 (8)	0.0124 (8)	−0.0010 (7)
C7	0.0235 (8)	0.0198 (8)	0.0227 (9)	−0.0035 (7)	0.0072 (7)	−0.0025 (6)
C8	0.0203 (8)	0.0242 (8)	0.0194 (9)	−0.0030 (7)	0.0049 (7)	−0.0021 (6)
C9	0.0219 (9)	0.0339 (10)	0.0318 (10)	0.0016 (8)	0.0120 (8)	−0.0040 (8)
C10	0.0181 (8)	0.0182 (8)	0.0200 (9)	−0.0017 (6)	0.0039 (6)	−0.0027 (6)
C11	0.0196 (8)	0.0207 (8)	0.0205 (9)	−0.0002 (6)	0.0051 (7)	0.0003 (6)
C12	0.0233 (9)	0.0260 (9)	0.0222 (9)	−0.0014 (7)	0.0058 (7)	0.0010 (7)
C13	0.0282 (9)	0.0324 (10)	0.0304 (10)	0.0071 (8)	0.0103 (8)	0.0106 (8)
C14	0.0219 (8)	0.0247 (8)	0.0198 (9)	−0.0015 (7)	0.0046 (7)	0.0006 (7)
C15	0.0355 (11)	0.0539 (13)	0.0387 (12)	0.0133 (10)	0.0218 (10)	0.0209 (10)
C16	0.0500 (16)	0.129 (3)	0.0391 (15)	0.0170 (17)	0.0182 (13)	0.0283 (16)
C17	0.0434 (13)	0.0516 (14)	0.086 (2)	0.0139 (11)	0.0349 (15)	0.0354 (14)
C18	0.042 (2)	0.0387 (19)	0.080 (3)	−0.0026 (16)	0.020 (2)	0.0083 (18)
C18'	0.079 (6)	0.126 (8)	0.058 (6)	0.012 (6)	0.021 (5)	0.010 (5)

### Geometric parameters (Å, °)

**Table:**

O1—C10	1.322 (2)	C10—C11	1.407 (2)
O1—H1	0.84	C11—C12	1.443 (2)
O2—C12	1.231 (2)	C11—C14	1.459 (2)
O3—C14	1.254 (2)	C12—C13	1.527 (2)
N1—C8	1.355 (2)	C13—H13A	0.99
N1—C7	1.387 (2)	C13—H13B	0.99
N1—C9	1.459 (2)	C15—C17	1.504 (3)
N2—C14	1.349 (2)	C15—C16	1.550 (3)
N2—C13	1.455 (2)	C15—H15	1.00
N2—C15	1.467 (2)	C16—C18'	1.525 (5)
C1—C8	1.390 (2)	C16—H16A	0.96
C1—C10	1.433 (2)	C16—H16B	0.96
C1—C2	1.450 (2)	C16—H16C	0.96
C2—C3	1.405 (2)	C16—H16D	0.96
C2—C7	1.406 (2)	C16—H16E	0.96
C3—C4	1.385 (3)	C17—C18	1.462 (4)
C3—H3	0.95	C17—H17A	0.96
C4—C5	1.393 (3)	C17—H17B	0.96
C4—H4	0.95	C17—H17C	0.96
C5—C6	1.384 (3)	C17—H17D	0.96
C5—H5	0.95	C17—H17E	0.96
C6—C7	1.398 (3)	C18—H18A	0.98
C6—H6	0.95	C18—H18B	0.98
C8—H8	0.95	C18—H18C	0.98
C9—H9A	0.98	C18'—H18D	0.98
C9—H9B	0.98	C18'—H18E	0.98
C9—H9C	0.98	C18'—H18F	0.98
			
C10—O1—H1	109.5	N2—C15—H15	107.6
C8—N1—C7	109.27 (14)	C17—C15—H15	107.6
C8—N1—C9	125.41 (16)	C16—C15—H15	107.6
C7—N1—C9	124.87 (15)	C18'—C16—C15	112.1 (5)
C14—N2—C13	111.38 (14)	C18'—C16—H16A	109.5
C14—N2—C15	123.56 (16)	C15—C16—H16A	109.6
C13—N2—C15	124.71 (15)	C15—C16—H16B	109.2
C8—C1—C10	128.05 (16)	H16A—C16—H16B	109.5
C8—C1—C2	106.28 (14)	C18'—C16—H16C	106.5
C10—C1—C2	125.66 (15)	C15—C16—H16C	109.6
C3—C2—C7	118.22 (15)	H16A—C16—H16C	109.5
C3—C2—C1	135.34 (15)	H16B—C16—H16C	109.5
C7—C2—C1	106.40 (15)	C18'—C16—H16D	109.7
C4—C3—C2	118.86 (17)	C15—C16—H16D	109.2
C4—C3—H3	120.6	H16B—C16—H16D	109.7
C2—C3—H3	120.6	H16C—C16—H16D	109.7
C3—C4—C5	121.56 (19)	C18'—C16—H16E	108.7
C3—C4—H4	119.2	C15—C16—H16E	109.2
C5—C4—H4	119.2	H16A—C16—H16E	107.6
C6—C5—C4	121.27 (17)	H16B—C16—H16E	111.7
C6—C5—H5	119.4	H16D—C16—H16E	107.8
C4—C5—H5	119.4	C18—C17—C15	113.6 (2)
C5—C6—C7	116.82 (17)	C18—C17—H17A	108.8
C5—C6—H6	121.6	C15—C17—H17A	109.0
C7—C6—H6	121.6	C18—C17—H17B	108.5
N1—C7—C6	128.67 (16)	C15—C17—H17B	109.1
N1—C7—C2	108.13 (14)	H17A—C17—H17B	107.6
C6—C7—C2	123.20 (17)	C15—C17—H17C	108.7
N1—C8—C1	109.93 (16)	H17A—C17—H17C	112.4
N1—C8—H8	125.0	H17B—C17—H17C	110.0
C1—C8—H8	125.0	C18—C17—H17D	107.9
N1—C9—H9A	109.5	C15—C17—H17D	110.0
N1—C9—H9B	109.5	H17A—C17—H17D	107.3
H9A—C9—H9B	109.5	H17C—C17—H17D	109.5
N1—C9—H9C	109.5	C18—C17—H17E	105.9
H9A—C9—H9C	109.5	C15—C17—H17E	109.8
H9B—C9—H9C	109.5	H17B—C17—H17E	109.7
O1—C10—C11	117.53 (14)	H17C—C17—H17E	109.5
O1—C10—C1	113.52 (15)	H17D—C17—H17E	109.5
C11—C10—C1	128.93 (15)	C17—C18—H18A	109.5
C10—C11—C12	133.64 (15)	H17C—C18—H18A	107.5
C10—C11—C14	118.53 (15)	C17—C18—H18B	109.5
C12—C11—C14	107.45 (14)	H17C—C18—H18B	118.4
O2—C12—C11	131.76 (16)	H18A—C18—H18B	109.5
O2—C12—C13	121.67 (16)	C17—C18—H18C	109.5
C11—C12—C13	106.56 (14)	H17C—C18—H18C	102.1
N2—C13—C12	104.50 (14)	H18A—C18—H18C	109.5
N2—C13—H13A	110.9	H18B—C18—H18C	109.5
C12—C13—H13A	110.9	C16—C18'—H18D	109.5
N2—C13—H13B	110.9	H16B—C18'—H18D	104.2
C12—C13—H13B	110.9	C16—C18'—H18E	109.5
H13A—C13—H13B	108.9	H16B—C18'—H18E	113.9
O3—C14—N2	124.21 (16)	H18D—C18'—H18E	109.5
O3—C14—C11	125.70 (16)	C16—C18'—H18F	109.5
N2—C14—C11	110.08 (15)	H16B—C18'—H18F	110.2
N2—C15—C17	111.19 (18)	H18D—C18'—H18F	109.5
N2—C15—C16	109.75 (18)	H18E—C18'—H18F	109.5
C17—C15—C16	112.9 (2)		
			
C8—C1—C2—C3	176.72 (18)	C1—C10—C11—C12	−5.6 (3)
C10—C1—C2—C3	−1.9 (3)	O1—C10—C11—C14	0.5 (2)
C8—C1—C2—C7	−0.62 (18)	C1—C10—C11—C14	−177.42 (16)
C10—C1—C2—C7	−179.19 (15)	C10—C11—C12—O2	5.9 (3)
C7—C2—C3—C4	−0.7 (3)	C14—C11—C12—O2	178.41 (19)
C1—C2—C3—C4	−177.76 (18)	C10—C11—C12—C13	−173.30 (18)
C2—C3—C4—C5	−1.6 (3)	C14—C11—C12—C13	−0.80 (19)
C3—C4—C5—C6	2.1 (3)	C14—N2—C13—C12	1.2 (2)
C4—C5—C6—C7	−0.4 (3)	C15—N2—C13—C12	174.59 (18)
C8—N1—C7—C6	180.00 (18)	O2—C12—C13—N2	−179.49 (17)
C9—N1—C7—C6	−7.3 (3)	C11—C12—C13—N2	−0.2 (2)
C8—N1—C7—C2	−0.17 (19)	C13—N2—C14—O3	177.45 (17)
C9—N1—C7—C2	172.49 (15)	C15—N2—C14—O3	4.0 (3)
C5—C6—C7—N1	177.90 (17)	C13—N2—C14—C11	−1.8 (2)
C5—C6—C7—C2	−1.9 (3)	C15—N2—C14—C11	−175.24 (17)
C3—C2—C7—N1	−177.39 (15)	C10—C11—C14—O3	−3.8 (3)
C1—C2—C7—N1	0.49 (18)	C12—C11—C14—O3	−177.61 (17)
C3—C2—C7—C6	2.5 (3)	C10—C11—C14—N2	175.43 (15)
C1—C2—C7—C6	−179.67 (16)	C12—C11—C14—N2	1.6 (2)
C7—N1—C8—C1	−0.24 (19)	C14—N2—C15—C17	115.6 (2)
C9—N1—C8—C1	−172.85 (15)	C13—N2—C15—C17	−57.0 (3)
C10—C1—C8—N1	179.06 (16)	C14—N2—C15—C16	−118.7 (2)
C2—C1—C8—N1	0.53 (18)	C13—N2—C15—C16	68.7 (3)
C8—C1—C10—O1	176.19 (15)	N2—C15—C16—C18'	177.0 (5)
C2—C1—C10—O1	−5.5 (2)	C17—C15—C16—C18'	−58.3 (6)
C8—C1—C10—C11	−5.8 (3)	N2—C15—C17—C18	−57.2 (3)
C2—C1—C10—C11	172.44 (16)	C16—C15—C17—C18	178.9 (2)
O1—C10—C11—C12	172.36 (17)		

### Hydrogen-bond geometry (Å, °)

**Table:**

*D*—H···*A*	*D*—H	H···*A*	*D*···*A*	*D*—H···*A*
O1—H1···O3	0.84	1.72	2.5003 (19)	154
C8—H8···O2	0.95	2.12	2.916 (2)	140
C9—H9C···O2^i^	0.98	2.51	3.441 (3)	159

Symmetry codes: (i) *x*, −*y*+3/2, *z*−1/2.

## References

[bb1] Allen, F. H., Kennard, O., Watson, D. G., Brammer, L., Orpen, A. G. & Taylor, R. (1987). *J. Chem. Soc. Perkin Trans. 2*, pp. S1–19.

[bb2] Baan, J. L. van der, Barnick, J. W. F. K. & Bickelhaupt, F. (1978). *Tetrahedron*, **34**, 223–231.

[bb3] Ellis, D. D. & Spek, A. L. (2001). *Acta Cryst.* C**57**, 433–434. 10.1107/s0108270100020758 11313585

[bb4] Holzapfel, C. W., Hutchison, R. D. & Wilkins, D. C. (1970). *Tetrahedron*, **26**, 5239–5246. 10.1016/s0040-4020(01)98733-2 5499899

[bb5] Mackellar, F. A., Grostic, M. F., Olson, E. C., Wnuk, R. J., Branfman, A. R. & Rinehart, K. L. Jr (1971). *J. Am. Chem. Soc.* **93**, 4943–4945. 10.1021/ja00748a067 5118218

[bb6] Matsuo, K., Kitaguchi, I., Takata, Y. & Tanaka, K. (1980). *Chem. Pharm. Bull.* **28**, 2494–2502. 10.1248/cpb.28.2494 7428128

[bb7] Rigaku (2005). *CrystalClear* Rigaku Corporation, Tokyo, Japan.

[bb8] Rinehart, K. L., Beck, J. R., Borders, D. B., Kinstle, T. H. & Krauss, D. (1963). *J. Am. Chem. Soc.* **85**, 4038–4039.

[bb9] Sheldrick, G. M. (2008). *Acta Cryst.* A**64**, 112–122. 10.1107/S0108767307043930 18156677

[bb10] Sticking, C. E. (1959). *Biochem. J.* **72**, 332–334.

[bb11] Wu, C.-S., Huang, J.-L., Sun, Y.-S. & Yang, D.-Y. (2002). *J. Med. Chem.* **45**, 2222–2228. 10.1021/jm010568y 12014960

